# Surgically Treated Clear Cell Sarcomas — What Influences Outcomes?

**DOI:** 10.1007/s13193-024-02108-4

**Published:** 2024-10-25

**Authors:** Harsha S. S. Tadala, Ashish Gulia, Ajay Puri, Bharat Rekhi, Siddhartha Laskar

**Affiliations:** 1 Orthopaedic Oncology, Department of Surgical Oncology, Homi Bhabha Cancer Hospital and Research Centre, Tata Memorial Centre, Visakhapatnam, India; 2 Surgical Oncology, Homi Bhabha Cancer Hospital and Research Centre, Tata Memorial Centre, New Chandigarh, Punjab India; 3 Department of Surgical Oncology, Tata Memorial Centre, HBNI, Mumbai, Maharashtra India; 4 Department of Pathology, Tata Memorial Hospital, Homi Bhabha National Institute (HBNI) University, Parel, Mumbai, Maharashtra 400012 India; 5 Department of Radiation Oncology, Tata Memorial Centre, HBNI, Mumbai, Maharashtra India

**Keywords:** Clear cell sarcoma, Metastasis

## Abstract

Clear cell sarcoma (CCS) also called as melanoma of soft parts is a rare malignant soft-tissue tumor with melanocytic differentiation, primarily located in deep soft tissue and has preference for lymph node and pulmonary metastasis. Metastatic patients have poor oncologic prognosis and so far, no adjuvant treatment seems to be effective in these cases. All cases were retrieved from our prospectively maintained surgical database. Twenty-nine patients (14 males,15 females) with a mean age of 34 years (13–69 years) were operated between 2004 and 2020. Overall survival and recurrence free survival were evaluated. At mean follow-up of 45 months, out of 29 cases, 2 lost to follow-up, 6 patients had nodal metastasis, 3 had lung metastasis, one had both at presentation, 15 patients had died due to disease and 12 are alive. Nodal metastasis rate was 24% (7/29). Positive margin is observed in 6 (19%). 24 patients had recurrence of which 6 had both local and distant. The 5-year OS, LRFS, DRFS were 53%, 56%, 35% respectively at 5 years. Survival rates were significantly poor in patients with metastasis at presentation, 66% vs. 25% (*p* = 0.016). CCS is an aggressive soft tissue malignancy with high propensity for metastasis. The overall survival of CCS is poor. Metastasis at presentation negatively impacts on survival. Distant recurrences, especially nodal recurrences are not uncommon and complete surgical resection of all recurrences if possible is advisable. Research to develop new chemotherapeutic agents and targeted therapies may help improve the prognosis of CCS.

## Introduction

Clear cell sarcoma (CCS) is a rare melanocytic soft tissue sarcoma (STS) which accounts for less than 1% of all soft tissue tumors [1]. It was described first by Enzinger in 1965 and shows a predilection for the deep soft tissues of the lower extremities close to the tendon, fascia, or aponeuroses [2]. These tumors originate from neural crest cells and are histologically characterized by clear cells that represent intracellular glycogen accumulation [3]. Although originally categorized by Enzinger as a STS, it has characteristics of both STS (deep soft tissue primary location, propensity for pulmonary metastasis) and melanoma (distal limb distribution, tendency for local recurrence, in-transit disease, and regional nodal spread) [4]. These tumors share histological and immunohistochemical characteristics with malignant melanoma and hence were initially described as “malignant melanoma of soft parts” [3]. Most CCS tumors are characterized by a recurrent chromosomal translocation, t(12; 22), resulting in fusion of the EWSR1gene on 22q12 with the ATF1 gene on 12q13 (3) which differentiates it from melanoma [5].

Wide surgical resection with negative margins forms the mainstay of treatment. Intralesional excision is associated with higher local failure rates. Though radiotherapy has a limited role in completely excised cases, it may improve outcomes in patients who undergo close resection margins [1]. The role of chemotherapy is very limited and is employed in patients with metastatic disease [1, 6]. Patients with metastasis have a poor oncologic prognosis. Literature regarding the treatment of CCS is limited due to its rarity.

In this study, we evaluated the oncological outcomes of surgically treated patients at our institution to determine possible prognostic factors.

## Materials and Methods

Between 2004 and 2020, 130 patients with the diagnosis of melanocytic tumors were retrieved from a prospectively maintained surgical database. 101 cutaneous melanomas and nodal lesions were excluded. Twenty-nine cases of histopathologically proven cases of clear cell sarcoma (CCS) were included in the study after ethics committee approval.

Clinical details, imaging, treatment records and post treatment surveillance status were retrieved from the medical records. Radiology was reviewed to confirm non-cutaneous location and histological specimens were re-evaluated to confirm the diagnosis of CCS. The diagnosis was based on morphological features of spindle cells arranged in sheets and nests separated by thick collagen bundles; tumor cells were oval to spindle with pleomorphic vesicular nuclei with prominent nucleoli with occasional giant cells. On immunohistochemistry, cells were positive for S100 and HMB 45, whereas EMA, SMA, CD34, BCL2 were negative in all cases (5).

Staging investigations included an ultrasound of the draining nodal basin and non-contrast computed tomography (CT) of thorax or a Positron emission tomography (PET) scan.

There were 15 females and 14 males with a mean age of 34 years (13–69 years). Upper limb was involved in nine patients and lower limb in 20 with thigh being the most common site of affection. Ten patients (35%) were metastatic at presentation. Six patients had isolated nodal, 3 had pulmonary and one patient had both nodal and pulmonary metastasis. Overall nodal metastasis rate was 24% (7/29).

Magnetic resonance imaging (MRI) was used for surgical planning in all cases. Complete surgical removal was the primary goal of surgery even if it necessitated ablating procedure like amputation. Lymph node dissection/pulmonary metastectomy was done in cases with metastatic disease to achieve R0 status. Pre-operative radiation was used if the tumor was close to vital structures to obtain tumor shrinkage to facilitate limb salvage. Lesions > 5 cm in size, inadvertent prior surgery and margin positive resection were given post operative radiation including brachytherapy. Decision of brachytherapy was based on intra operative evaluation by radiotherapist, if there was a layer of soft tissue/fascia covering vital structures like vessels, nerve and bone.

Twenty-five patients underwent limb salvage surgery and 4 had amputation. Five of 25 limb salvage surgeries were scar excisions because of prior inadvertent excisions elsewhere. Five patients had axillary nodal dissection and 2 had pelvic node dissection. Of the 3 patients with lung lesions, 2 patients with small, sub centimeter lesions were kept under active surveillance and 1 patient underwent metastectomy. The patient with both, pulmonary and nodal (axillary nodes) metastasis underwent metastectomy and nodal dissection along with resection of primary lesion.

Patients followed-up every 6 months after treatment for the first 5 years, followed by annual visits for the next 5 years. Follow-up evaluation included clinical examination and a local ultrasound and chest radiograph. Additional cross-sectional imaging for the operative site, nodal basin and lungs was only done on suspicion of local or distant recurrence on clinical examination and chest radiographs.

Local recurrence free, distant recurrence free and overall survivals were calculated from date of surgery to date of recurrence or death from any cause. Survival curves were computed according to Kaplan–Meier method. A Cox proportional hazard model was used to identify variables influencing survival.

## Results

Size was available in 26 patients; three patients were scar excisions. The mean size of the resected lesions was 5.5 cm (2–15 cm). Five patients required additional soft tissue cover for wound closure. The average nodal yield was 3 (range = 1–4). Six patients had microscopic margin positive resection, and all received post-operative radiation. Overall, 22 patients received radiation (2 — pre-operative radiation, 2 — brachytherapy, 18 — post-operative radiation). None of the patients received chemotherapy.

Two patients were lost to follow-up at 7 and 35 months, respectively. At a mean follow-up of 45 months (12–118 months), 15 patients have died and 12 are alive.

### Local Recurrence (all patients)

Six of 27 patients developed local recurrence at a mean of 36 months (15–58 months). Three patients had isolated local recurrence and underwent surgical excision, all developed unresectable distant metastasis. Two succumbed to disease at 52 and 58 months from index surgery and one is on supportive care. Three patients developed both local and pulmonary relapse (19–50 months). Of these 3, two underwent surgery at both sites and are alive at last follow-up (46 and 108 months). One patient had disseminated disease and was treated with best supportive care.

Of 6 margin positive resections, only one patient developed local recurrence. He underwent a wide excision of the recurrence but developed multiple lung metastasis 8 years from index surgery. He is currently alive with the disease. The other five developed distant recurrences and only one patient is alive after subsequent surgical resection.

The local recurrence free survival of the entire cohort was 88% and 56% at 3 years and 5 years, respectively. It was significantly worse in cases with upper limb lesions compared to lower limb lesions (31% vs. 67% *p* = 0.04) (Table 1).

**Table 1:** Prognostic

Prognostic Factor	N	OS	LRFS
Age			
< 30	15	54.2	50.8
> 30	14	53.7	87.5
Site			
Lower limb	20	54.1	66.7
Upper limb	9	50.8	31.2(p-0.04, CI-95%)
Size			
< 5 cm	13	63.5	54
> 5 cm	13	33.8	75
Margin			
Positive	6	22.2	-
Negative	23	60.9	60.8
RT			
Yes	22	55.9	52.8
No	7	42.9	66.7
Metastatic at presentation			
Yes	10	25.0(0.016)	-
No	19	66.3	-

### Distant Recurrences

#### Non-metastatic at Presentation

Of 19 non-metastatic patients, one was lost to follow-up at 35 months. Fourteen developed distant recurrence (2–64 months, mean: 28 months). Various sites of recurrence were nodes (8), lungs (4), brain (1), bone (1), and soft tissue (3). Three of these patients had distant recurrence at two sites synchronously. Of the 14, nine were treated surgically. Six of 9 patients are alive at last follow-up (27–107 months), two died due to disease progression and one was lost to follow-up. In the other five, complete surgical resection at all sites was not possible and they eventually died due to disease.

#### Metastatic at Presentation

Ten patients were metastatic at presentation: 3 had lung metastasis, 6 had nodal metastasis, and 1 had both. Of the 3 patients who had isolated lung metastasis, one patient underwent metastectomy along with resection of primary lesion but subsequently developed recurrence in the lung 4 months after index surgery and died 11 months later. Two underwent only resection of primary lesion and were kept under surveillance, in view of small size of the lung lesions. In both patients, pulmonary lesions progressed on follow-up. One patient had unresectable disease and died after 10 months and another patient underwent metastectomy and developed multiple lung metastasis 8 years from index surgery and is currently alive with the disease.

The patient with both, pulmonary and nodal (axillary nodes) metastasis underwent metastectomy and nodal dissection along with resection of primary lesion and developed recurrence in the lung after 10 months and died of the disease 15 months post index surgery.

All six patients with isolated nodal metastasis underwent nodal dissection along with resection of primary lesion. Of these, one had recurrence in the lung after 1 year, underwent metastectomy and is alive after 10 years of follow-up. Five patients developed unresectable recurrences at various sites (3–23 months) and died of disease progression (12–36 months).

Only 1 out of 10 patients metastatic at presentation are disease-free and alive at last follow-up.

### All Patients

5-year local recurrence free survival for patients who received radiation was 52% vs 67% (*p* = 0.49).

The mean time after surgery till recurrence was 20 months, ranging from 2 to 64 months (27 months for non-metastatic patients, 17 months for patients metastatic at presentation).

Twenty-four patients had distant recurrences at a mean of 22 months (3–64 months) (lung metastasis in 6, bone in 1, brain in 1, only nodal in 9, soft tissue in 1, both lung and nodal in 2, lung and soft tissue in 2, nodal and soft tissue in 1, soft tissue lung and bone in 1). Of 24 distant recurrences, 12 patients underwent surgical resection, the rest were all treated with best supportive care due to extensive disease. Of 12 patients who had surgical resection 3 patients died. Thus, 15 of 24 patients died after distant recurrence. The distant recurrence free survival of the entire cohort was 41% and 35% at 3 years and 5 years, respectively. 5-year survival after surgical resection of recurrence was 65% as compared to 20% (*p* = 0.016) in those that did not have resection.

**Fig. 1:**
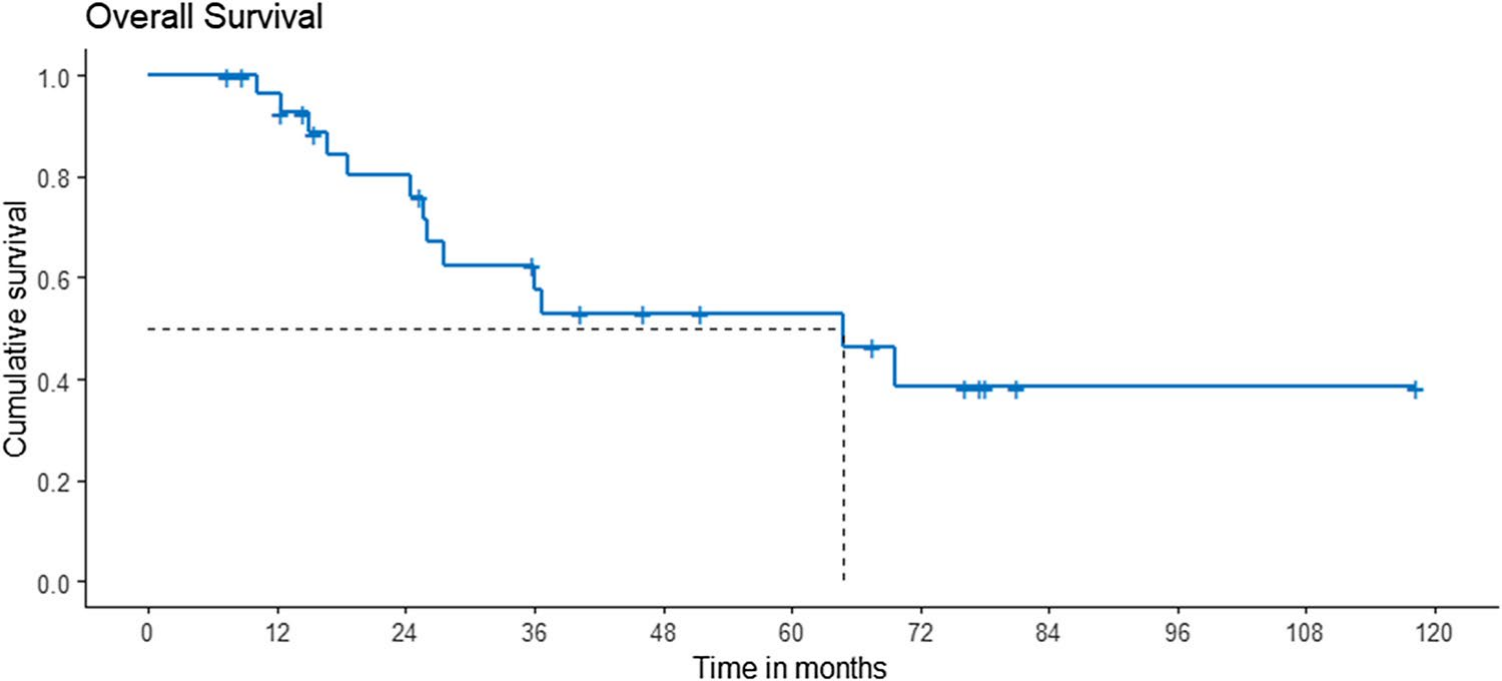
Overall survival of entire cohort in this series

**Fig. 2:**
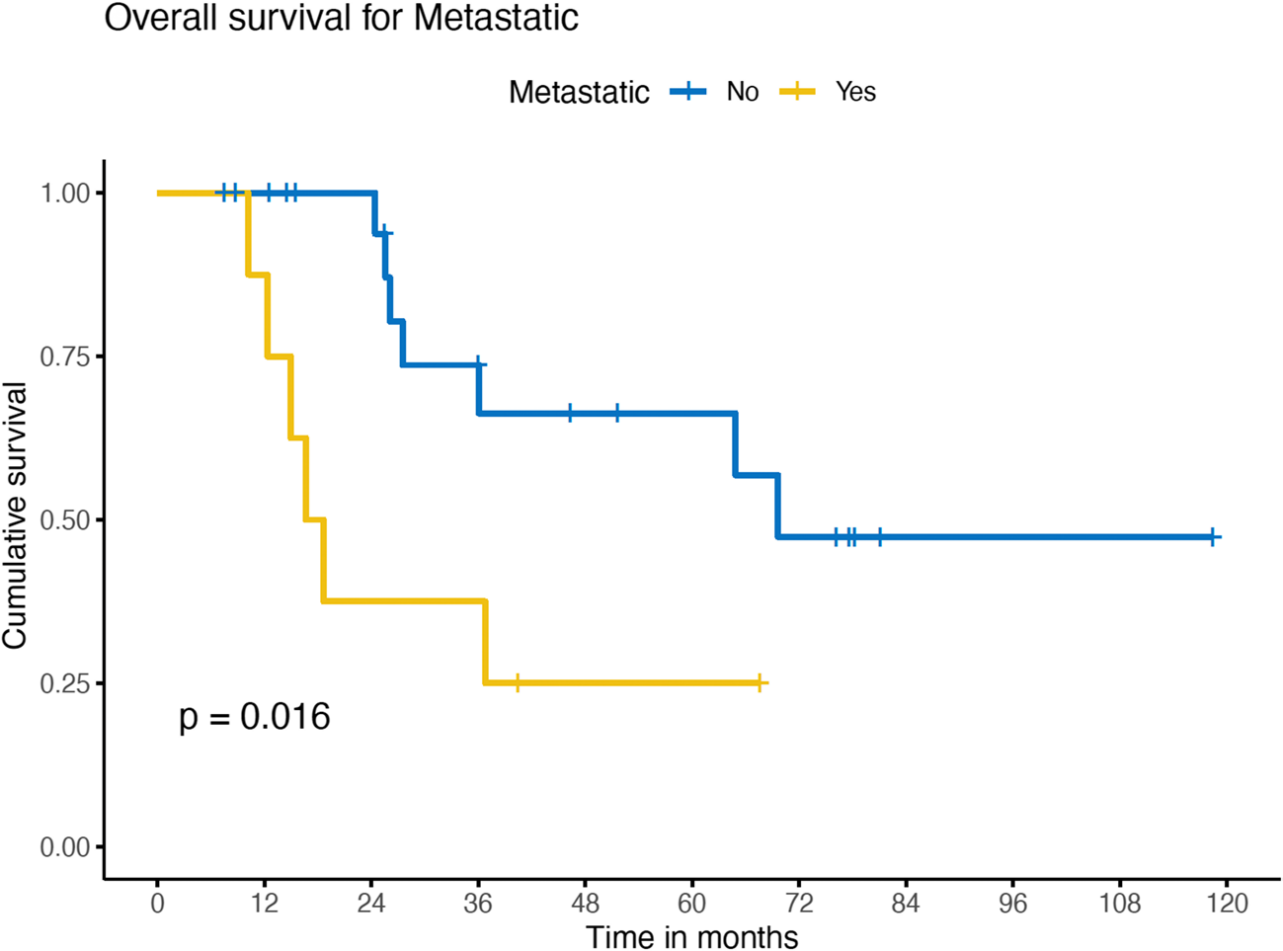
Overall survival – Metastatic vs Non metastatic in this series

The overall survival of the entire cohort was 63% and 53% at 3 years and 5 years, respectively (Fig. 1). Survival rates were significantly poorer in patients with metastasis at presentation, 66% vs. 25% (*p* = 0.016) (Fig. 2).

Patients with a tumor less than 5 cm had a 5-year overall survival of 64% as against 34% for those with a tumor greater than 5 cm (*p* = 0.34).

The event free survival of the entire cohort was 39% and 14% at 3 years and 5 years, respectively. Event free survival for metastatic patients at presentation was 0%, compared to 19% in non-metastatic patients (*p* = 0.05).

## Discussion

Enzinger first described CCS, a rare soft tissue sarcoma in 1965 (4). It presents as a slowly growing painless firm mass involving the aponeuroses, tendons and fascial structures [2, 7]. It is more common in young adults, between the ages of 20 and 40 years [3]. The mean age in our study was 34 years, ranging from 13 to 69 years which is similar to published studies [3, 7, 8].

Over half of the cases reported in the literature are less than 5 cm [2, 7–9]. In our study too, 50% of our cases were less than 5 cm in size. Our study showed a trend toward better survival for patients who had tumors less than 5 cm (5 years OS 34% for > 5 cm vs. 64% for < 5 cm). Hocar et al. in their study of 52 patients, showed that tumor size is the only significant prognostic factor on multivariate analysis [3]. Bianchi et al. also reported a better 5-year disease-free survival of 83% for tumors < 5 cm vs. 25% for tumors > 5 cm (7).

Though radiotherapy is used in the adjuvant setting in most series, the survival advantage is not clear in literature [8, 10]. In our institute too, CSS is treated on the principles of soft tissue sarcoma. Hence, we routinely use radiotherapy as an adjuvant to surgery. 22 of 29 patients received radiotherapy in our study, but our data did not demonstrate any benefit in local control to those given radiotherapy [52% vs 67% (*p* = 0.49)]. It could be argued, as radiotherapy was given to patients with poorer prognostic factors (lesions > 5 cm in size, inadvertent prior surgery and margin positive resection), it helped achieve similar local control in these lesions.

Being a chemo resistant histology, the role of chemotherapy in CCS is limited. We did not use chemotherapy and hence are unable to comment on its efficacy. There have been reports with small numbers that have shown a benefit with the use of various agents (tyrosine kinase inhibitors, caffeine potentiated chemotherapy regimen) in both, primary and relapsed cases [11–13].

CCS, unlike most soft tissue sarcomas, has a propensity for lymph node metastasis [2, 4]. These are observed in 12–53% in patients with clear cell sarcoma, when compared to 2–6% with soft tissue sarcomas [2, 7, 11]. 24% (7/29) of patients had lymph node metastasis at presentation in our study. This may not be a true reflection of nodal metastasis as our study only includes patients that had surgical treatment. Patients with nodal metastasis as a part of multiorgan unresectable disease were treated non-surgically with supportive care and hence excluded from our study.

Even though CCS has a high propensity for lymph node metastasis, the usefulness of sentinel lymph node biopsy and elective node dissection is not well established [3, 15]. We undertake nodal dissection only in the presence of nodal metastasis confirmed by fine-needle aspiration cytology. Eight of 19 (42%) non metastatic patients developed subsequent nodal recurrence. It is a matter of conjecture whether sentinel lymph node biopsy would have identified these nodes at index presentation. Further research in the form of large multicenter trails is warranted to establish the use of sentinel lymph node biopsy or elective nodal dissection in CCS. Having said that, only one of 6 patients with isolated nodal metastasis in our study is alive. Thus, it is debatable if identification of covert nodal metastasis by sentinel node biopsy earlier will impact on survival. CCS is associated with a high rate of local recurrence, distant recurrences, and regional lymph node recurrences [8, 9]. Irrespective of their metastatic status at presentation, more than two-third of patients in our study developed relapses (23 patients) after initial surgical treatment. In our study, nodal recurrence was observed in 11 patients (38%) and pulmonary metastasis in 10 patients (34%). The mean time to lymph nodal metastasis was shorter than for metastasis at other sites (8 months vs 32 months). Bianchi et al. [7] also reported early lymph node recurrence at a median time of 11 months. CCS demonstrates a high incidence of nodal recurrence on follow-up unlike soft tissue sarcomas (< 10% nodal recurrence) [12, 13] while the incidence of pulmonary recurrences is comparable (30%) [14]. This pattern of metastasis in CCS suggests that its behavior is more akin to melanoma despite its soft tissue origin [3, 7, 8].

Of 24 patients with distant recurrence, 12 had complete surgical excision of the recurrences. Of these 12, 8 patients are alive at last follow-up, indicating the importance of complete surgical removal in recurrences. Only three patients of 11 with nodal recurrences are alive.

With a 5-year overall survival of 53%, metastasis at presentation is the only prognostic factor in our study which impacted on overall survival (66% vs. 25%) (Table 1, Fig. 2). This is similar to other studies where survival ranged from 47 to 67% [8, 15–17]. Tumor size > 5 cm had a tendency for poorer survival but failed to reach statistical significance in our study, though other authors have mentioned the prognostic value of size [8, 18].

In our study, we observed local recurrences as late as 5 years and distant recurrences even after 5 years. Hence, long-term follow-up is advisable in CCS. Early detection and prompt surgical excision of recurrences may help improve survival. Clark et al. [8] and Hocar et al. [3] also suggested long-term follow-up for late local recurrences and metastasis. Apart from pulmonary and nodal metastasis, we also observed distant recurrences in soft tissues (5), bone (2) and brain. Most of these were associated with lung or nodal lesions, hence it may be prudent to recommend whole-body screening investigations if any metastatic foci are identified on surveillance imaging.

Our study suffers from the inherent limitations of any retrospective study. The relatively small number of cases is inevitable in a single institution study for a rare sarcoma. The strength of our study is that all patients were treated by the same multidisciplinary team, maintaining consistency in decision making and protocols. Our experience adds to the relatively sparse data on these uncommon lesions and may help guide colleagues in their decisions.

## Conclusion

CCS is an aggressive soft tissue malignancy with high propensity for metastasis. The overall survival of CCS is poor. Metastasis at presentation negatively impacts on survival. While lesions > 5 cm tend to have worse survival, the use of radiotherapy as an adjuvant may help local control in such lesions. Distant recurrences, especially nodal recurrences are not uncommon and complete surgical resection of all recurrences if possible is advisable. Research to develop new chemotherapeutic agents and targeted therapies may help improve the prognosis of CCS.

## References

[1] Mavrogenis AF, Bianchi G, Stavropoulos NA, Papagelopoulos PJ, Ruggieri P (2013) Clinicopathological features, diagnosis and treatment of clear cell sarcoma/melanoma of soft parts. Hippokratia 17(4):298–302 25031505 PMC4097407

[2] Chung EB, Enzinger FM (1983) Malignant melanoma of soft parts. A reassessment of clear cell sarcoma. Am J Surg Pathol 7(5):405–13 6614306 10.1097/00000478-198307000-00003

[3] Hocar O, Le Cesne A, Berissi S, Terrier P, Bonvalot S, Vanel D et al (2012) Clear cell sarcoma (malignant melanoma) of soft parts: A clinicopathologic study of 52 cases. Dermatol Res Pract 2012:1–8 10.1155/2012/984096 PMC3369396 22693489

[4] Enzinger FM (1965) Clear-cell sarcoma of tendons and aponeuroses. An analysis of 21 cases. Cancer 18(9):1163–74 14332545 10.1002/1097-0142(196509)18:9<1163::aid-cncr2820180916>3.0.co;2-0

[5] Humbert, Richard; David A. Adler, Christine M. Disteche, Christopher Hassett, Curtis J. Omiecinski CEF, Orr H, Chung M yi, Banfi S, Kwiatkowski T, Servadio A et al (1993) © 199 3 Nature Publishing Group http://www.nature.com/naturegenetics. Nat Genet 3:73–96

[6] Deenik W, Mooi WJ, Rutgers EJ, Peterse JL, Hart AA, Kroon BB (1999) Clear cell sarcoma (malignant melanoma) of soft parts: A clinicopathologic study of 30 cases. Cancer 86(6):969–975 10491522

[7] Bianchi G, Charoenlap C, Cocchi S, Rani N, Campagnoni S, Righi A et al (2014) Clear cell sarcoma of soft tissue: a retrospective review and analysis of 31 cases treated at Istituto Ortopedico Rizzoli. Eur J Surg Oncol 40(5):505–10. 10.1016/j.ejso.2014.01.016 24560887 10.1016/j.ejso.2014.01.016

[8] Clark MA, Johnson MB, Thway K, Fisher C, Thomas JM, Hayes AJ (2008) Clear cell sarcoma (melanoma of soft parts): The Royal Marsden Hospital experience. Eur J Surg Oncol 34(7):800–804 18042498 10.1016/j.ejso.2007.10.006

[9] Kawai A, Hosono A, Nakayama R, Matsumine A, Matsumoto S, Ueda T et al (2007) Clear cell sarcoma of tendons and aponeuroses: A study of 75 patients. Cancer 109(1):109–116 17133413 10.1002/cncr.22380

[10] Deenik W, Mooi WJ, Rutgers EJ, Peterse JL, Hart AAKBB (1999) Clear cell sarcoma (malignant melanoma) of soft parts: a clinicopathologic study of 30 cases. Cancer 15 86(6):9 10491522

[11] Clear cell sarcoma. A clinicopathologic study of 27 cases - PubMed [Internet]. [cited 2021 Jun 3]. Available from: https://pubmed.ncbi.nlm.nih.gov/6616410/

[12] Fong Y, Coit DG, Woodruff JM, Brennan MF (1993) Lymph node metastasis from soft tissue sarcoma in adults: Analysis of data from a prospective database of 1772 sarcoma patients. Ann Surg 217(1):72–77 8424704 10.1097/00000658-199301000-00012 PMC1242736

[13] Riad S, Griffin AM, Liberman B, Blackstein ME, Catton CN, Kandel RA et al (2004) Lymph node metastasis in soft tissue sarcoma in an extremity. In: Clinical Orthopaedics and Related Research. Lippincott Williams and Wilkins 129–34 10.1097/01.blo.0000141660.05125.46 15346063

[14] Billingsley KG, Burt ME, Jara E, Ginsberg RJ, Woodruff JM, Leung DHY et al (1999) Pulmonary metastases from soft tissue sarcoma: Analysis of patterns of disease and postmetastasis survival. In: Annals of Surgery. Ann Surg 602–12 10.1097/00000658-199905000-00002 PMC1420804 10235518

[15] William Finley J, Hanypsiak B, Mcgrath B, Kraybill W, Gibbs JF (2001) Clear cell sarcoma: the roswell park experience. J Surg Oncol 77(1):16–20 11344475 10.1002/jso.1057

[16] Lucas DR, Nascimento AG, Sim FH (1992) Clear cell sarcoma of soft tissues: mayo clinic experience with 35 cases. Am J Surg Pathol 16(12):1197–1204 1463095 10.1097/00000478-199212000-00006

[17] Deenik W, Mooi WJ, Rutgers EJ, Peterse JL, Hart AA, Kroon BB, et al (1999) Clear Cell Sarcoma (Malignant Melanoma) of Soft Parts A Clinicopathologic Study of 30 Cases 10491522

[18] Kawai A, Hosono A, Nakayama R, Matsumine A, Matsumoto S, Ueda T, et al. Clear cell sarcoma of tendons and aponeuroses: A study of 75 patients. Cancer [Internet]. 2007 Jan 1 [cited 2021 Jun 4];109(1):109–16. Available from: https://pubmed.ncbi.nlm.nih.gov/17133413/10.1002/cncr.22380 17133413

